# Hydatids in Bone

**Published:** 1870-09

**Authors:** 


### Hydatids in Bone.

At a meeting of the Berlin Medical Society, Dr. Kuster related, with full details, a case of echinococci in bone. The patient was a laborer, of twenty-two, who fractured his humerus, about the middle of the shaft, when twelve years old; good union took place. About ten years afterwards he was knocked down by a bull, and suffered a fracture of the same bone, just above the condyles. A plaster-of-Paris apparatus proved of no avail, a second was not more successful, and he was sent to the hospital. The surgeons at first considered that the former bandages had not been sufficiently tight; rubbing of the ends of the fragments was effected, and the arm and shoulder well secured. This first dressing turned out fruitless, as also did two subsequent ones; and Dr. Wilms, who had charge of the case, resolved to use ivory pegs. The gimlet, with the slightest effort, sank freely into the bone, and *pus* issued from the opening thus made. The idea of inserting pegs was therefore given up, and the arm kept quiet. Feverishness and severe swelling ensued, and Dr. Kuster was obliged to make two lateral incissions, which freed some pus and peculiar-looking membranous shreds. These turned out to be portions of hydatid cysts. A drainage tube was then inserted, and on each dressing vessels of echinococci were discharged, varying in size from a flax seed to a goose's egg. This escape of hydatids lasted about four weeks, when both echinococci and pus disappeared. The limb was now attacked with erysipelas, at the termination of which the ends of the fragments remained bathed in a sanious fluid. Soon afterwards the elbow joint inflamed, and unmistakable symptoms of pyæmia set in. Dr. Kuster now disarticulated the limb at the shoulder-joint. On an examination of the part, an extensive purulent infiltration of the intermuscular cellular tissue, up to the shoulder, was discovered. A large sac, full of pus, surrounded the ends of the ununited fragments. In this pus, cysts, varying in size from that of a cherry to a nut, floated about. In the surrounding muscles, about twenty small cysts were detected, some the size of a pin's head, others as laıge as peas, and everywhere a communication with the principal purulent sac could be made out. The author states that no case of hydatids in *muscles* have been recorded since Dupuytren observed them in a case of hydatids of the humerus. On making a longitudinal section of the bone, the medullary substance was found to have been destroyed, a complete vacuum being observed in its stead. Such a case shows plainly how we may be far from the mark when we attribute non-union to some defect in the constitution, to malposition, to imperfect treatment, to a piece of membrane or muscle between the fragments, &c., &c. Dr. Kuster has taken the trouble to collect the cases of a similar nature scattered in books and periodicals. He finds only 21 or 22, one case being doubtful. Tibia, 5; cranium, 4 (1 doubtful); spinal column, 3; pelvis, 3; humerus, 3; femur, 2; phalanx of finger, 1. In five there was fracture; non-union, of course, ensued, but there had mostly been some swelling of the bone, which broke on the slightest provocation. In most cases there were no symptoms leading to the suspicion of hydatids. The case of Messrs. Dickenson and Crompton is quoted (where is it consigned ?) where a girl broke her humerus by a fall on the staircase; non-union was treated by a seton, and the pus contained hydatids. (Could not the latter have been found in the pus independently of the tracture?—*Med. Centr. Zeitung*, April 22nd, 1870).—*Lancet.*

